# Supplementary material for: A numerical approach for detecting switch-like bistability in mass action chemical reaction networks with conservation laws
Source: BMC Bioinformatics. 2022 Jan 4;23:1. doi: 10.1186/s12859-021-04477-x (PMC8725470; doi:10.1186/s12859-021-04477-x)
Supplement: Supplementary file 1 — Additional file 1. Deriving a Conservation Law Matrix (B) with Non-Negative Integer Values. [file 12859_2021_4477_MOESM1_ESM.pdf]

# Deriving a B Matrix with Non-Negative Integer Values

August 29, 2021

## 1 Introduction

This supplementary document provides a demonstration of **Algorithm 1** from the main text. The objective of this algorithm is to derive a matrix B that represents material (chemical moiety) conservation laws in a conventional form, where elements are stoichiometry coefficients (i.e. non-negative integers). By definition, we have that the $B$ matrix is the nullspace of $S^T$, however, simply finding an arbitrary nullspace is likely to result in basis vectors with fractional numbers that span both negative and positive values. Below we demonstrate how Algorithm 1, when applied to the futile cycle and prion coupled with double phosphorylation models, outlined in the main text, produces a $B$ matrix with non-negative integer values.

## 2 Futile cycle

Below we provide the Stoichiometric matrix for the futile cycle system shown in Figure 4 of the main text.

$$S = \begin{array}{c} \\ S \\ E1 \\ E1S \\ S^* \\ E2 \\ E2S \end{array} \begin{array}{c} \begin{array}{ccccccccccc} r_1 & r_2 & r_3 & r_4 & r_5 & r_6 & r_7 & r_8 & r_9 & r_{10} & r_{11} \end{array} \\ \begin{pmatrix} -1 & 1 & 0 & -1 & 1 & 0 & 0 & 0 & 0 & 0 & 1 \\ -1 & 1 & 1 & 0 & 0 & 0 & -1 & 1 & 0 & 0 & 0 \\ 1 & -1 & -1 & 0 & 0 & 0 & 0 & 0 & -1 & 1 & 0 \\ 0 & 0 & 1 & 0 & 0 & 1 & 0 & 0 & 0 & 0 & -1 \\ 0 & 0 & 0 & -1 & 1 & 1 & 1 & -1 & 0 & 0 & 0 \\ 0 & 0 & 0 & 1 & -1 & -1 & 0 & 0 & 1 & -1 & 0 \end{pmatrix} \end{array}$$

Theoretically, there is an infinite number of nullspace basis vectors for the above matrix. The actual choice of the nullspace basis vectors depends on the underlying algorithm of a computation tool. When using the Python library SymPy, we obtain:

$$\tilde{B} = Null(S^T) = span\left\{ \begin{bmatrix} -1 \\ 1 \\ 0 \\ -1 \\ 1 \\ 0 \end{bmatrix}, \begin{bmatrix} 1 \\ 0 \\ 1 \\ 1 \\ 0 \\ 1 \end{bmatrix} \right\} = span\left\{ \tilde{\mathbf{b}}_1, \tilde{\mathbf{b}}_2 \right\}$$

From the derived $Null(S^T)$, we see that the dimension of the nullspace is two ($\lambda = 2$), thus there are two conservation laws.

From the above example, we see that the nullspace basis vectors may contain negative values. Additionally, the nullspace can contain fractional numbers. Although these nullspaces are mathematically correct, the interpretation of such vectors as chemical moiety conservation laws is non-intuitive. Ideally, they should be composed of non-negative integers.

Our first step is to find another set of basis vectors that are non-negative. To begin, we consider a linear combination of the basis vectors using the vector $\mathbf{x} = (x_1, x_2)$ as coefficients. This new basis vector is denoted as $\mathbf{z} = x_1\tilde{\mathbf{b}}_1 + x_2\tilde{\mathbf{b}}_2 = \tilde{\mathbf{B}} \cdot \mathbf{x}$ in the main text. This linear combination will form a new basis vector $\mathbf{z}$ and we would like this basis vector to have only positive entries, thus, we require that $\mathbf{z} \geq \mathbf{0}$. Finding $\mathbf{x}$ such that $\mathbf{z} \geq \mathbf{0}$ can be formulated as a linear programming problem. Note, that although constructing $\mathbf{z} \geq \mathbf{0}$ results in 6 inequalities, only the following 3 of them are not redundant. The following set of inequalities corresponds to the non-redundant row entries of the expression $-\tilde{B} \cdot \mathbf{x} \leq \mathbf{0}$ of the optimization problem (3) of the main text.

$$\begin{cases} -x_1 + x_2 \geq 0 \\ x_1 \geq 0 \\ x_2 \geq 0 \end{cases} \tag{1}$$

Note that we are guaranteed to have a non-negative basis vector because chemical conservation laws are expressed with non-negative integer values. From the inspection of the system of inequalities (1), one can see that there are an infinite number of solutions (e.g. if both $x_1$ and $x_2$ are scaled by a constant factor). To constrain the number of solutions, we choose an arbitrary scaling factor that makes $\sum_{i=1}^{N} z_i = 1$. This equality constraint corresponds to the expression $\left( \sum_{i=1}^{N} \tilde{B}_{i,1}, \ldots, \sum_{i=1}^{N} \tilde{B}_{i,\lambda} \right)^T \mathbf{x} = 1$ in optimization problem (3) of the main text.

$$0x_1 + 4x_2 = 1 \tag{2}$$

In Figure S1 we present a visual representation of the inequality constraints (1) and equality constraint (2). Solving this linear optimization problem results in two unique $\mathbf{x}$ vectors: $(0, 0.25)$ and $(0.25, 0.25)$. Using these found vectors, one can transform the originally found nullspace basis vectors, which results in the non-negative basis vectors.

$$Null(S^T) = span\left\{ \begin{bmatrix} 0 \\ 0.25 \\ 0.25 \\ 0 \\ 0.25 \\ 0.25 \end{bmatrix}, \begin{bmatrix} 0.25 \\ 0 \\ 0.25 \\ 0.25 \\ 0 \\ 0.25 \end{bmatrix} \right\}$$

The next of objective is to obtain a nullspace basis with a non-negative integer form. To accomplish this, we scale the $\mathbf{z}$ basis vectors by dividing by $min(\mathbf{z})$, where $min(\mathbf{z}) \neq 0$. This

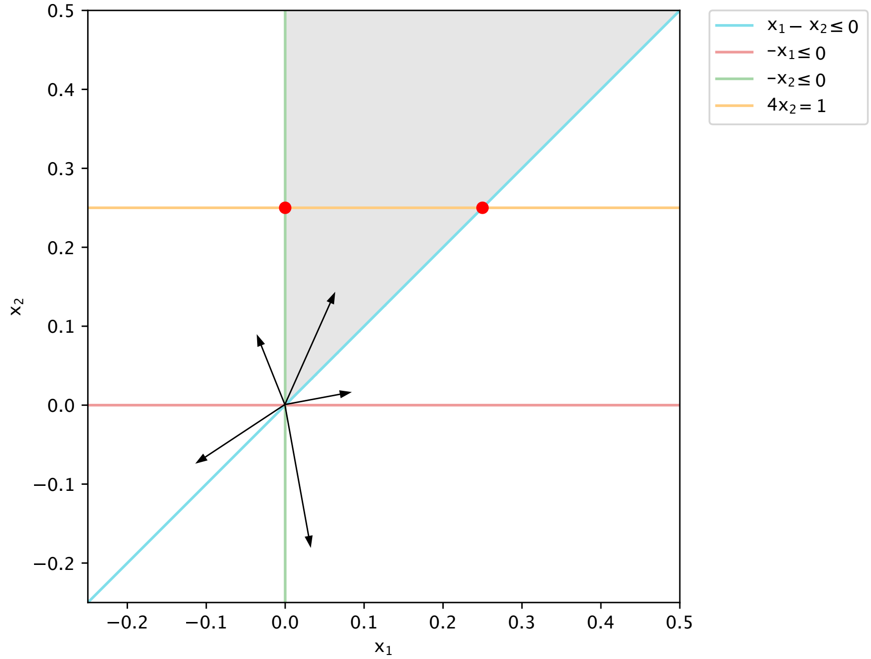


**Supplementary Fig. 1:** A visual representation of the optimization problem for finding the non-negative nullspace basis vectors. The shaded area are the solutions satisfying the inequality system (1). The orange line constrains the solutions such that the sum of the elements of **z** equals 1. The arrows coming out of the point $(0, 0)$ correspond to the vectors **w** and indicate the random search directions for the optimization problem. Although any point on the orange line within the shaded area satisfies the system of inequalities and the equality constraint, the Simplex optimization algorithm ensures that only vertices are considered. Thus, effectively reducing the number of unique solutions to $\lambda = N - rank(S)$ (the number of conservation laws). Points labeled as red dots correspond to the **x** vector solutions that produce the new non-negative nullspace basis vector ($\mathbf{z} = \tilde{B} \cdot \mathbf{x}$).

results in the desired nullspace.

$$ker(S^T) = span\left\{\begin{bmatrix}0\\1\\1\\0\\1\\1\end{bmatrix}, \begin{bmatrix}1\\0\\1\\1\\0\\1\end{bmatrix}\right\}$$

Note, dividing by the smallest positive number does not always guarantee that we will produce a basis vector with integer elements. For example, this can be seen in the case where we have an equilibrium between two oligomers with a prime number of subunits. Lets, say $A_3$ contains 3 subunits of A and $A_7$ contains 7 subunits of A. The corresponding conservation law appears as follows: $C_1 = 7A_3 + 3A_7$. Its obvious that reducing the smallest coefficient (at $A_7$) to 1, results in a non-integer coefficient for $A_7$. However, this approach will work for the vast majority of cases where the conservation law involves molecules

with no more than one of the same chemical moiety (e.g. $C_1 = 7A + A_7$). Moreover, there is nothing crucial about expressing the conservation law in integer form for the algorithm. The integer form that is desired is purely for convenience of human readability. The evaluation of bistability does not require that the conservation laws are expressed with integer coefficients.

The aforenoted integer nullspace basis vectors, given the order of the species (rows) in the stoichiometry matrix **S**, the conservation laws can be re-written as:

$$C_1 = c_2 + c_3 + c_5 + c_6$$
$$C_2 = c_1 + c_3 + c_4 + c_6$$

or using chemical species notation.

$$E_{tot} = [E1] + [E1S] + [E2] + [E2S]$$
$$S_{tot} = [S] + [E1S] + [S^*] + [E2S]$$

## 3 Prion/Double Phosphorylation

Below we illustrate the same process of finding the chemical moiety conservation laws from the stoichiometry matrix for the Prion/Double Phosphorylation model depicted in Figure 6 of the main text. The Stoichiometric matrix is as follows.

$$S = \begin{array}{c} \\ E1 \\ E2 \\ E1E2 \\ E2E1 \\ S \\ S^* \\ SE2 \\ S^{**} \\ S^*E2 \end{array} \begin{array}{c} \begin{array}{cccccccccccccc} r_1 & r_2 & r_3 & r_4 & r_5 & r_6 & r_7 & r_8 & r_9 & r_{10} & r_{11} & r_{12} & r_{13} & r_{14} \end{array} \\ \begin{pmatrix} -1 & 1 & -1 & 1 & 2 & 0 & 0 & 0 & 0 & 0 & 0 & 0 & 0 & 0 \\ -1 & 1 & -1 & 1 & 0 & 2 & -1 & 1 & 1 & 0 & -1 & 1 & 1 & 0 \\ 1 & -1 & 0 & 0 & -1 & 0 & 0 & 0 & 0 & 0 & 0 & 0 & 0 & 0 \\ 0 & 0 & 1 & -1 & 0 & -1 & 0 & 0 & 0 & 0 & 0 & 0 & 0 & 0 \\ 0 & 0 & 0 & 0 & 0 & 0 & -1 & 1 & 0 & 1 & 0 & 0 & 0 & 0 \\ 0 & 0 & 0 & 0 & 0 & 0 & 0 & 0 & 1 & -1 & -1 & 1 & 0 & 1 \\ 0 & 0 & 0 & 0 & 0 & 0 & 1 & -1 & -1 & 0 & 0 & 0 & 0 & 0 \\ 0 & 0 & 0 & 0 & 0 & 0 & 0 & 0 & 0 & 0 & 0 & 0 & 1 & -1 \\ 0 & 0 & 0 & 0 & 0 & 0 & 0 & 0 & 0 & 0 & 1 & -1 & -1 & 0 \end{pmatrix} \end{array}$$

This matrix provides the corresponding nullspace, which was obtain from SymPy:

$$Null(S^T) = span\left\{ \begin{bmatrix} -1 \\ -1 \\ -2 \\ -2 \\ 1 \\ 1 \\ 0 \\ 1 \\ 0 \end{bmatrix}, \begin{bmatrix} 1 \\ 1 \\ 2 \\ 2 \\ 0 \\ 0 \\ 1 \\ 0 \\ 1 \end{bmatrix} \right\}.$$

As shown before, to find non-negative basis vectors we have to find the coefficients of the linear combination defined by the vector $\mathbf{x} = (x_1, x_2)$. These coefficients must satisfy

the following non-redundant inequalities.

$$\begin{cases} -x_1 + x_2 \geq 0 \\ x_1 \geq 0 \\ x_2 \geq 0 \end{cases} \tag{3}$$

As before, to limit the number of solutions we add an equality constraint: $\sum_{i=1}^{N} z_i = 1$.

$$3x_1 + 8x_2 = 1 \tag{4}$$

A graphical representation of the system of three inequalities (3) and equality (4) is show in Figure S2.

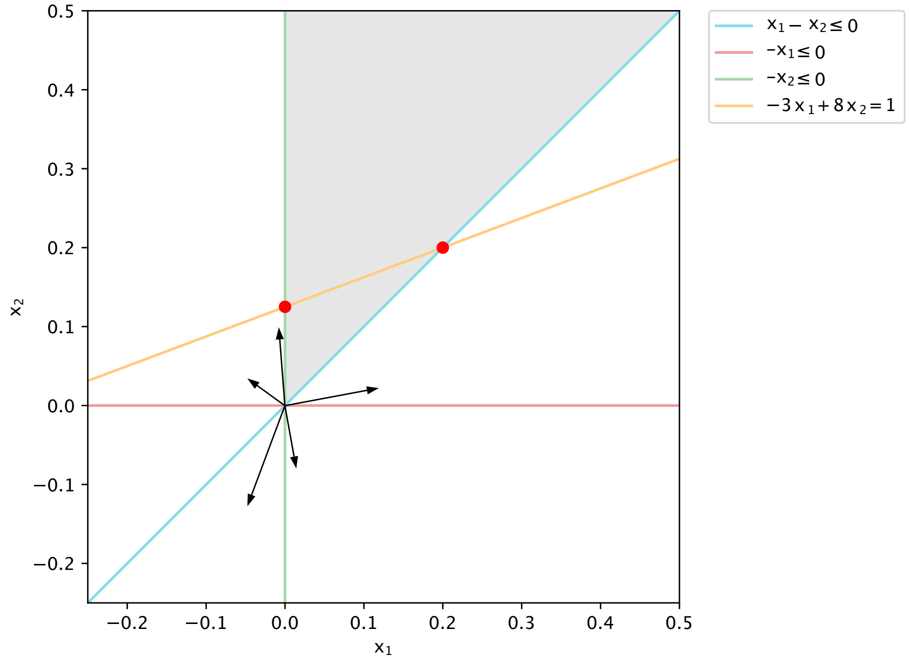


**Supplementary Fig. 2:** A visual representation of the optimization problem for finding a non-negative nullspace for the prion/double phosphorylation model. For details on notation, see Figure S1

Solving the linear optimization problem results in two unique **x** vectors: (0, 0.125) and (0.2, 0.2). Transforming the originally found nullspace basis vectors results in the non-

negative basis vectors.

$$Null(S^T) = span\left\{\begin{bmatrix} 0 \\ 0 \\ 0 \\ 0 \\ 0.2 \\ 0.2 \\ 0.2 \\ 0.2 \\ 0.2 \end{bmatrix}, \begin{bmatrix} 0.125 \\ 0.125 \\ 0.25 \\ 0.25 \\ 0 \\ 0 \\ 0.125 \\ 0 \\ 0.125 \end{bmatrix}\right\}$$

Dividing each basis by the smallest positive number results in the following basis vectors.

$$Null(S^T) = span\left\{ \begin{bmatrix} 0 \\ 0 \\ 0 \\ 0 \\ 1 \\ 1 \\ 1 \\ 1 \\ 1 \end{bmatrix}, \begin{bmatrix} 1 \\ 1 \\ 2 \\ 2 \\ 0 \\ 0 \\ 1 \\ 0 \\ 1 \end{bmatrix} \right\}$$

These provided basis vectors can be re-written as the following chemical moiety conservation laws.

$$C_1 = c_5 + c_6 + c_7 + c_8 + c_9$$
$$C_2 = c_1 + c_2 + 2c_3 + 2c_4 + c_7 + c_9$$

Alternatively, using chemical species notation the conservation laws look as follows.

$$S_{tot} = [S] + [S^*] + [SE2] + [S^{**}] + [S^*E2]$$
$$E_{tot} = [E1] + [E2] + 2[E1E2] + 2[E2E1] + [SE2] + [S^*E2]$$
